# Genetic structure of coral-*Symbiodinium* symbioses on the world’s warmest reefs

**DOI:** 10.1371/journal.pone.0180169

**Published:** 2017-06-30

**Authors:** Edward G. Smith, Benjamin C. C. Hume, Patrice Delaney, Jörg Wiedenmann, John A. Burt

**Affiliations:** 1 Marine Biology Laboratory, CGSB, New York University Abu Dhabi, Abu Dhabi, United Arab Emirates; 2 Coral Reef Laboratory, University of Southampton/National Oceanography Centre, Southampton, United Kingdom; Department of Agriculture and Water Resources, AUSTRALIA

## Abstract

Corals in the Arabian/Persian Gulf (PAG) survive extreme sea temperatures (summer mean: >34°C), and it is unclear whether these corals have genetically adapted or physiologically acclimated to these conditions. In order to elucidate the processes involved in the thermal tolerance of PAG corals, it is essential to understand the connectivity between reefs within and outside of the PAG. To this end, this study set out to investigate the genetic structure of the coral, *Platygyra daedalea*, and its symbiotic algae in the PAG and neighbouring Gulf of Oman. Using nuclear markers (the ITS region and an intron of the Pax-C gene), this study demonstrates genetic divergence of *P*. *daedalea* on reefs within the thermally extreme PAG compared with those in the neighbouring Gulf of Oman. Isolation by distance of *P*. *daedalea* was supported by the ITS dataset but not the Pax-C intron. In addition, the symbiont community within the PAG was dominated by C3 symbionts, while the purportedly thermotolerant clade D was extremely rare and was common only at sites outside of the PAG. Analysis of the psbA^ncr^ indicates that the C3 variant hosted by *P*. *daedalea* in the PAG belongs to the newly described species, *Symbiodinium thermophilum*. The structuring of the coral and symbiont populations suggests that both partners of the symbiosis may contribute to the high bleaching thresholds of PAG corals. While limited gene flow has likely played a role in local adaptation within the PAG, it also indicates limited potential for natural export of thermal tolerance traits to reefs elsewhere in the Indian Ocean threatened by climate change.

## Introduction

Coral reefs have undergone global decline in recent decades, often as a result of bleaching events, where a breakdown of the symbiosis between the coral hosts and their algal partners is associated with declines in coral health and survival. Mass bleaching typically occurs under elevated sea temperatures, with increases of only 1–2°C above the normal maximum often resulting in widespread mortality of corals throughout the tropics [1]. Given that tropical sea temperatures are predicted to rise by 0.5–4°C by the end of the century [2], the maintenance of present-day coral reefs will depend on the ability of the holobiont to acclimate or adapt to future temperature increases.

Both the algal symbiont and the coral host have been proposed to play a role in thermal tolerance. Different strains of algal symbionts vary in their sensitivity to thermal stress, with thermally tolerant clades typically common in high-temperature coral habitats [3, 4]. With regard to the host, studies have demonstrated that differences in host thermal tolerance are correlated with genetic divergence and differences in gene and protein expression under different thermal regimes [5, 6]. In reality, both partners in the symbiosis likely play an interactive role in the thermal tolerance of the holobiont [7], suggesting that studies on thermal adaptation must consider the symbiont and host genotypes.

Corals that exist in contemporary extreme environments represent useful models for understanding the potential for acclimation or adaptation to future temperature increases [8]. Corals in the Arabian/Persian Gulf (hereafter ‘the PAG’) are exposed to extreme summer sea temperatures compared with other tropical regions, with temperatures exceeding 34°C for several months annually and summer maxima >36°C [9–11], and therefore have the potential to provide important insights into how corals respond to thermal stress. Although numerous studies have demonstrated that the PAG is biogeographically unique in terms of community structure (e.g. [12]), few studies have explored the molecular mechanisms that allow corals to persist in this environment. Given that PAG reefs are relatively geographically isolated from the Indian Ocean by the narrow (42 km wide) Strait of Hormuz, yet are relatively young (present-day shorelines reached ~6000 years ago [13]), it is unclear whether there has been sufficient isolation to support genetic adaptation to the local environment. The aim of this study was to assess the genetic structure of both the coral host and the symbiotic algae of the locally abundant and pan-tropically distributed coral, *Platygyra daedalea*. By comparing the genetic structure of the holobiont collected from sites within the PAG, with those in the neighbouring Gulf of Oman (including the Straits of Hormuz), where temperatures are more benign (mean monthly maximum <31.5°C), this study aims to elucidate whether differences in thermal tolerance are linked to genetic divergence in either the host or symbionts. Such findings would have important implications regarding the potential for natural exchange of thermal tolerance traits with Indian Ocean reefs threatened by future climate change.

## Results & discussion

**Fig 1:**
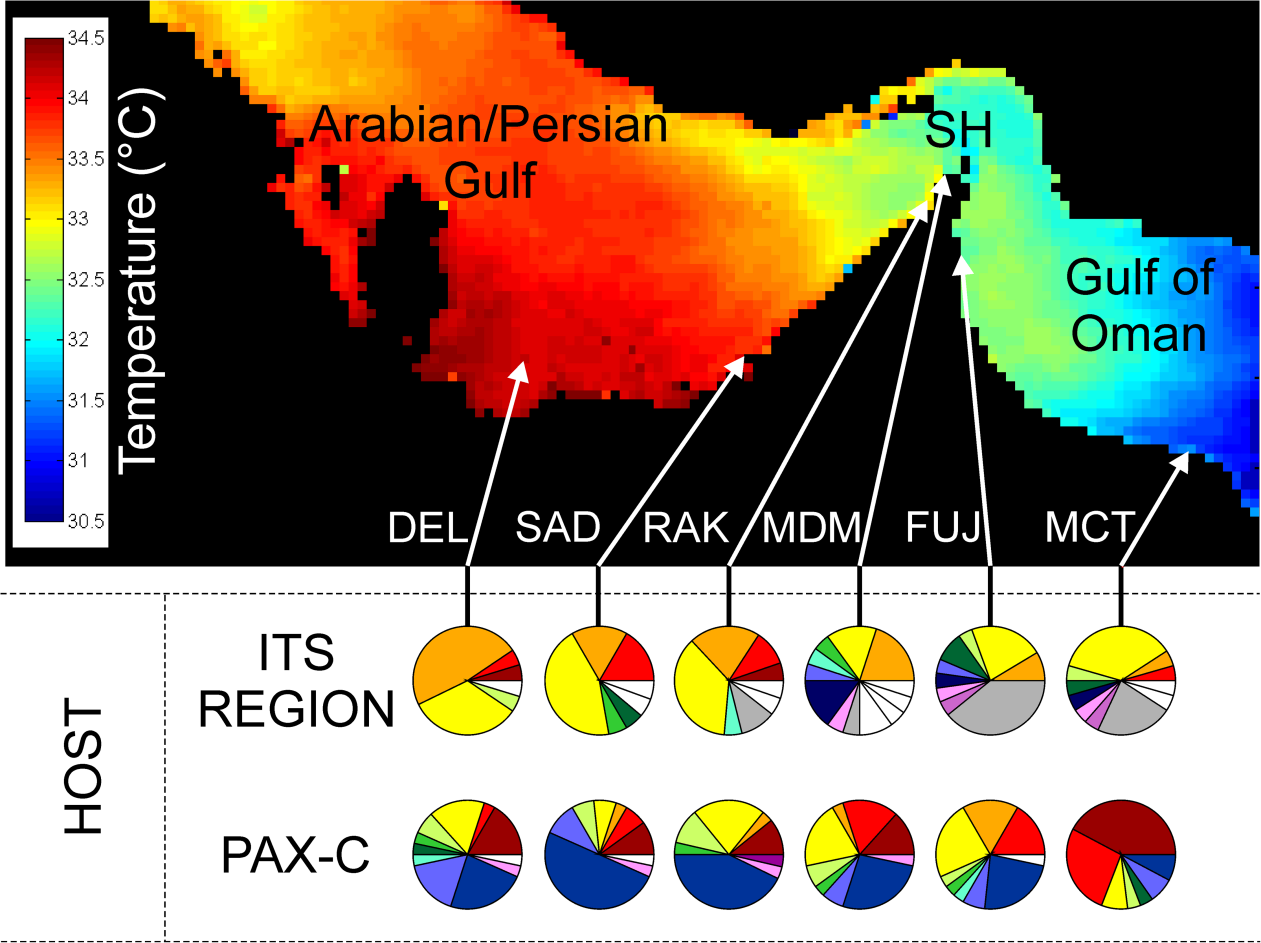
Genetic structure of *Platygyra daedalea* in the PAG and Gulf of Oman. *Upper panel*: Mean monthly averaged maximum SST, 2004–2014. *Lower panel*: Host haplotype frequencies. Each colour represents a different haplotype and white sectors indicate private haplotypes. SH = Strait of Hormuz.

The role of the host in the thermal tolerance of PAG holobionts has, to date, been unexplored. The results presented here from the host ITS marker indicate that there is strong genetic structuring between local populations in the PAG and Gulf of Oman. The haplotype frequencies (Fig 1) and pairwise ɸ_ST_ comparisons (Table 1) from the ITS marker demonstrate differentiation between subpopulations from the southern PAG (Delma and Saadiyat) and the Gulf of Oman (Fujairah and Muscat) and revealed isolation by distance (IBD) occurring in this system (Mantel test, r = 0.60, p = 0.026). As differences between the PAG and Gulf of Oman could arise from the presence of cryptic species, haplowebs were used to visualise “fields for recombination” (FFRs) [14, 15] (S1 and S2 Figs). This analysis did not reveal the presence of cryptic species in our dataset. The reduced gene flow observed between conspecific *Platygyra* populations in the southern PAG and Gulf of Oman has important implications, particularly as the ITS marker has previously shown panmixia of coral populations over a comparatively much larger scale of thousands of kilometres [16]. As limited gene flow promotes local adaptation [17], the strong genetic structuring observed in this marker supports the assertion that these coral hosts have adapted to the PAG’s extreme environment [18]. Nevertheless, such restricted gene flow suggests that substantial direct export of the acquired thermal tolerance traits of *P*. *daedalea* from the southern PAG to the wider Indian Ocean is unlikely. As a consequence, PAG corals may have a limited capacity to naturally enhance the resilience of Indian Ocean reefs threatened by future temperature increases associated with climate change.

**Table 1:** Pairwise ɸ_ST_ comparisons between sites for the ITS region (*below diagonal*) and PAX-C intron (*above diagonal*).

	DEL	SAD	RAK	MDM	FUJ	MCT
DEL		-0.010	**0.051**	-0.023	0.016	0.051
SAD	0.051		0.021	-0.014	0.035	**0.132**
RAK	0.052	0.008		**0.052**	**0.120**	**0.239**
MDM	**0.124**	**0.113**	0.026		-0.007	**0.087**
FUJ	**0.297**	**0.260**	**0.105**	**0.071**		**0.150**
MCT	**0.230**	**0.172**	0.038	0.034	-0.027	

Bold values indicate significance at p<0.05 level.

In agreement with the ITS marker, the results from the host Pax-C intron also indicated some structuring between sites inside the PAG and those in the Gulf of Oman, although there are some inconsistencies in the overall pattern. While there was agreement with structuring between the southern-most Gulf of Oman sites (Fujairah and Muscat) and the more proximal PAG sites (Ras al Khaimah and Saadiyat), it was not evident between the two most distant sites in this study (Muscat versus Delma). This was not consistent with the geography and oceanography of the region. Consequently, IBD is not evident from the PAX-C dataset (Mantel test, r = -0.17, p = 0.323). It was postulated that the discrepancies resulted from this type of marker, since nuclear intron markers such as Pax-C have frequently failed to resolve patterns observed in morphological features and other genetic markers, including the ITS region (e.g. [19, 20]). Although mitochondrial markers could provide an alternative to nuclear intron markers, they typically lack the resolution for intraspecific studies due to the slow evolution of the mitochondrial genome in anthozoans [21, 22]. The incongruence between the markers highlights the limitations of traditional markers as population genetic parameters vary across the genome. However, more representative estimates can now be obtained using approaches such as RADseq, which benefit from genome-wide marker coverage.

**Fig 2:**
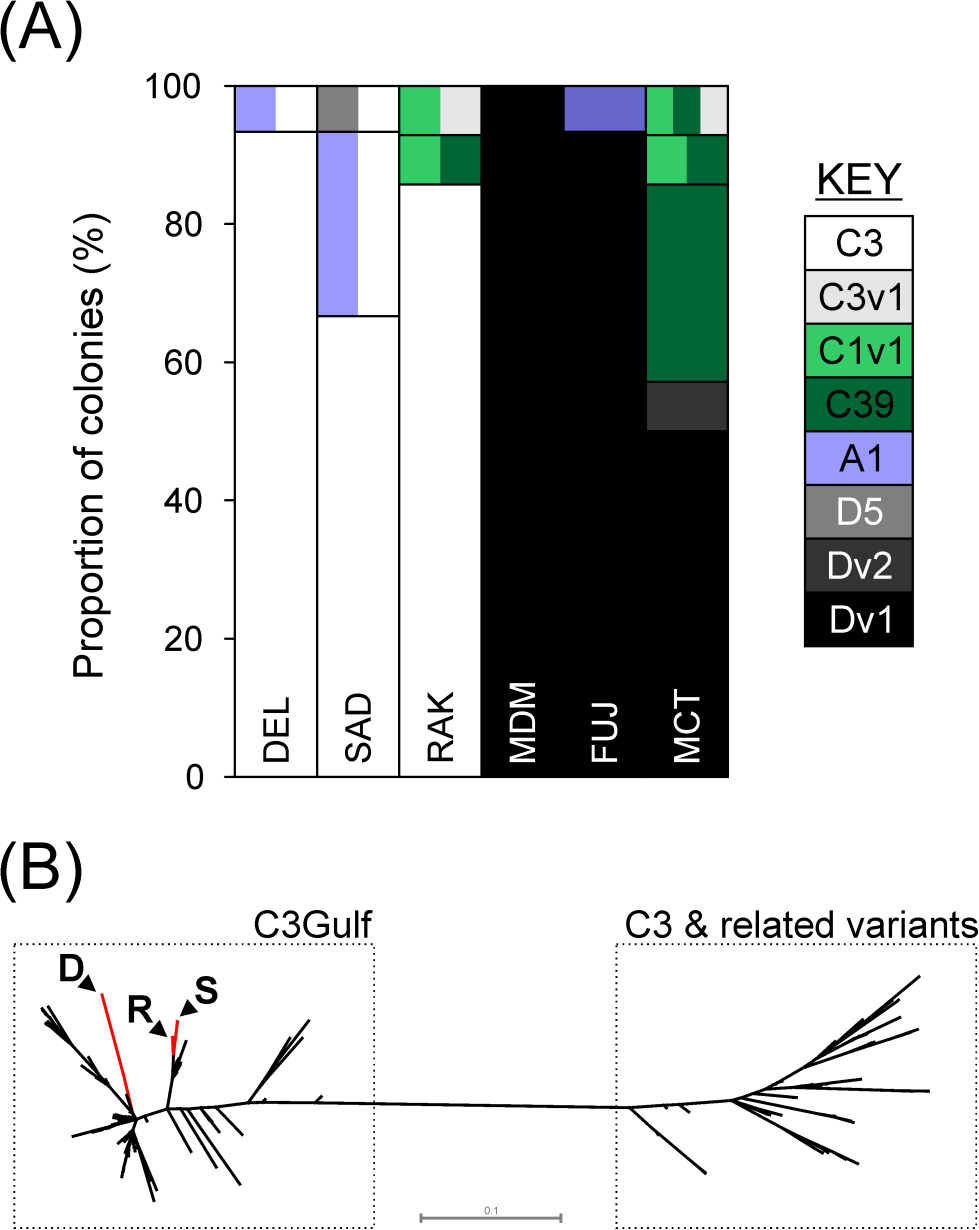
Characterisation of symbiont communities in the southern PAG and Gulf of Oman. (A) Bar charts indicate the proportion of colonies hosting each ITS2 type or combination of ITS2 types. Vertical separation within the bars indicates colonies where more than one symbiont type was present. (B) Radial phylogenetic tree depicting the relationships between C3 variants found in *P*. *daedalea* from PAG reefs, C3 variants from *Porites spp*. within the region and other C3 variants found elsewhere. The tree was generated by Bayesian inference analysis of the psbA^ncr^ region using the alignment generated in a previous study [18]. The branches containing samples from this study are shown in red with labelled arrows indicating the location of the samples (D: Delma; S: Saadiyat; R: Ras al Khaimah). The accession numbers for samples used to generate the tree are shown in S3 and S4 Tables.

The symbiont communities associated with *P*. *daedalea* showed clear structuring (Fig 2A). There were significant differences in the abundances of different ITS2 types hosted by corals (Fisher-Freeman-Halton Exact, p = 0.000) between the PAG and Gulf of Oman. Corals from the Gulf of Oman were largely dominated by clade D derivatives whereas corals from the PAG were associated with C3 symbionts. These results were unusual as it would be expected that clade D symbionts, widely regarded as thermotolerant, would be abundant in the world’s warmest reefs, and since previous work has shown that clade D symbionts become more dominant over C3 types in *Platygyra* populations as temperature increases along a thermal gradient in Taiwan [23]. In north-eastern Arabian reefs, *Platygyra* populations tend to be dominated by either D variants or C3 symbionts. Clade D dominated populations associate with the more benign environments such as the Gulf of Oman, Iranian and Saudi Arabian reefs [3, 4], while the more thermally extreme reefs of the southern PAG host C3 symbionts [10, 18, 24–27]. The distinct D or C3 dominated communities were clearly exemplified by the sharp switch from D dominated communities in the Musandam peninsula, Strait of Hormuz, to C3 dominated reef (Ras Al Khaimah) that is situated only 35km away. Although Musandam and Ras Al Khaimah are close and experience similar summer thermal maxima, they are situated at the transition between the Gulf of Oman and the PAG and therefore, environmental variables other than thermal maxima, such as salinity [28] may be influencing the community structure. The clear separation of D and C3 communities contrasts the gradient observed in *Porites* symbioses [18], where there is a more gradual transition between the two symbiont types. As *Porites* spp. are vertical transmitters, there is the potential for transport of symbionts with the host larvae and this may increase the dispersal potential of C3 in *Porites* spp, relative to the C3 in the horizontally transmitting *P*. *daedalea*. The clear boundary between the C3 and D dominated communities in *P*. *daedalea* is in contrast to the IBD pattern observed in the host ITS marker and could reflect different processes affecting the distribution of the coral larvae and the algal symbionts.

The ITS2 type C3 found in the PAG have recently been described as a new species [25], *Symbiodinium thermophilum*, which is genetically distinct from other C3 variants, and explains the unusual occurrence of a purportedly sensitive symbiont in the thermal extreme PAG [24]. Analysis of the psbA^ncr^ sequences from the *P*. *daedalea* C3 symbionts in the Gulf demonstrated they are more closely related to *S*. *thermophilum* than other C3 variants, and revealed further diversity within this newly described lineage (Fig 2B). The apparent ubiquity of *S*. *thermophilum* among species in the southern PAG supports previous assertions that it may be important in the persistence of corals in this region [25, 26].

The differences observed in the structuring of coral-symbioses in the PAG and Gulf of Oman are likely to be driven by the oceanographic conditions. While the extremes in salinity and summer temperatures exert strong selective pressures on the corals and their symbionts, the circulation patterns may also impact the population structuring and connectivity. As *P*. *daedalea* is a broadcast spawner, dispersal will be strongly driven by hydrodynamic conditions. The water entering the PAG travels as a surface current and proceeds north along the Iranian coast, while the outflow from the PAG travels as a subsurface current [29]. The reduced direct mixing of Gulf of Oman waters with the southern PAG, subsurface outflow preventing transport of buoyant larvae, and long residence times of PAG waters (>3 years; [30]) may limit exchange between corals outside and inside the Gulf. Nevertheless, studies on *Diadema setosum* have shown comparatively lower structuring (Fst = 0.05) between PAG and Gulf of Oman sites than was observed for the *P*. *daedalea* ITS marker [31]. Furthermore, the circulation patterns evident at the Strait of Hormuz may help explain why the Musandam and Ras Al Khaimah symbiont communities are distinct, as the Musandam reef is strongly influenced by the Gulf of Oman whereas Ras Al Khaimah is found outside of the inflow limits [29].

Considering the additional thermal stress associated with predicted rising sea surface temperatures, there is increasing interest in understanding the processes that lead to survival of corals in existing extreme reefs. The data presented here suggest that survival in the world’s warmest reefs likely involves both partners of the symbiosis. Previous work has documented responses associated largely with only one of the partners [5, 6, 23]. However, the ITS data indicates that *P*. *daedalea* in the PAG exhibits structuring of both the host population and the symbiont communities, although further work is required to ascertain the relative contribution of the different environmental factors to the structuring. While temperature and salinity are major factors controlling the coral community composition in the north-eastern Arabian peninsula, other environmental factors such as chlorophyll *a* concentrations have an influence [12] and could also impact corals and their symbionts at the population scale. Nevertheless, the data presented here provide a platform on which the genomic basis of thermal tolerance and responses to other environmental factors can be explored in both the host and symbiont.

## Materials and methods

### Host and symbiont markers

*P*. *daedalea* was selected for this study as it is widespread throughout the tropics and is a dominant member of the PAG’s coral communities [32]. Small fragments (1cm^3^, 14–15 individuals per site) of *P*. *daedalea* were collected at <8m depth from six reefs (DEL, Delma; SAD, Saadiyat; RAK, Ras al Khaimah; MDM, Musandam; FUJ, Fujairah; MCT, Muscat) ranging from Western Abu Dhabi to Muscat in May and June 2013 (Fig 1; S1 Table). Samples were placed into ziplock bags and immediately transferred to dry ice, with long-term storage at -80°C.

The coral host was analysed using two nuclear markers, the ITS region of the nrDNA and a Pax-C intron, while the symbiont communities were characterised using DGGE analysis of the ITS2 marker. DNA was extracted using a CTAB phenol-chloroform protocol [33]. Both host and symbiont markers were amplified using the primers and PCR cycling conditions outlined in S2 Table. All PCRs were performed using the Advantage 2 HF Taq, with the exception of the psbA^ncr^, and were purified using Qiagen PCR purification kits. The psbA^ncr^ marker was amplified using the PrimerStar GXL enzyme. The sequencing of the host and psbA^ncr^ markers were performed by Bioneer, South Korea, using the PCR primers. Sequences used in this study are available in GenBank (S4 Table).

Sequence chromatograms were visually inspected, secondary peaks called and manually checked in Sequencher 5.1. Double peaks were observed in 43% of the ITS region sequences, indicative of heterozygosity, as observed in other coral genera [19, 34]. As the chromatograms contained at most two sequences, they were processed under the framework used by Flot and coworkers [34]. One indel was common at the end of ITS sequences, but as it was at the end of our sequence alignment, it did not require deconvolution. Three individuals did possess length-variant heterozygote (LVH) sequences associated with single base indels and individual haplotypes were recovered from forward and reverse chromatograms using the software Champuru [35]. Indelligent [36] was used to recover the phase information from the remaining 30bp that were not covered by the reverse chromatogram. Haplotypes from the other heterozygous individuals were resolved statistically using PHASE [37] implemented using SeqPHASE [38]. The chromatograms for three individuals with phasing posterior probabilities below 0.9 were inspected and in two individuals, the haplotypes were confirmed based on differences in peak height at heterozygous positions. As the haplotypes for the remaining individual could not be determined unambiguously, we removed this individual from the ITS dataset. Both haplotypes from heterozygous individuals were included in the downstream population genetic analyses [19].

The PAX-C intron contained two common indel sites of 3bp and 4bp, separated by 360bp. As such, 60% of the sequences contained numerous double peaks indicative of length variant heterozygotes (LVHs). Forward and reverse sequences from LVHs were deconvoluted using Champuru [35]. The remaining sequences were phased using SeqPHASE and PHASE [37], with the haplotypes from the LVHs used as known haplotypes. The haplotypes for one sample could not be determined unambiguously and this sample was therefore not included in the analyses.

Pairwise ɸ_ST_ comparisons (F_ST_ calculated using sequence divergence) and Mantel tests were calculated in WinArl 3.5.1.2 [39]. Haplotype networks were constructed in Network 5.0 and turned into “haplowebs” using Inkscape. Haplotype frequencies for both markers are shown in S5 and S6 Tables.

### Symbiont community DGGE analyses

The DGGE analyses performed here follow the protocol outlined by Hume and coworkers [25]. Briefly, for all samples, the complete ITS1-5.8S-ITS2 region of the symbiont nrDNA was amplified using the primers and PCR conditions li0073ted in S2 Table. The resulting products were subsequently run on a 0.8% agarose gel and excised with a scalpel. The excised bands were homogenised in 500μl of deionised water using a micropestle. The ITS2 amplification with DGGE clamp was performed as per S2 Table using the homogenate as the template.

The DGGE analyses were performed on a BioRad DCode system with a model 475 gradient former. 10μl of PCR product was mixed with 10μl of 2x loading dye, and loaded into an 8% gel with a 32.5–57.5% gradient. The gel was run for ~1400Vh. Using the 16 well comb, 14 samples were loaded per gel, with 4 reference ladders. DGGE profiles that were identified as novel (i.e. had not been characterised previously) were first validated as genuine ITS2 sequence variants as opposed to structural conformations (as per [25]) and were subsequently cloned using the Strataclone kit, according to the manufacturers protocol, and plasmids sequenced by a commercial provider (Eurofins). Sequences were cropped and local BLAST performed against the KAUST ITS2 database [40] to avoid conflicting assignments from different sources within GenBank. In this study, we observed novel banding patterns for three clade D types (compared to sequenced variant used in the ladder), ITS2 type C39 (S3 Fig) and C1v1 (1bp different from ITS2 type C1). The novel clade D types were characterised by a variant that contained one homoduplex (D5), while the other two variants (Dv1 and Dv2) contained more than one homoduplex. Dv1 was characterised by two homoduplexes (D5 and D6). The presence of multiple heteroduplexes in the banding pattern for clade D variant Dv2 (S3 Fig) indicates the presence of multiple ITS2 types of which some either co-migrant with other homoduplexes or are below the detection limit. Cloning of 12 ITS2 sequences from this individual revealed 7 unique ITS2 sequences that were within 2 substitutions from ITS2 types D1, D4 and D6 (3 ITS2 exact matches, 3 sequences with 1 mismatch, 1 sequence with 2 substitutions). As the DGGE fingerprints are sufficient for distinguishing symbiont types [41], determining the exact composition of dominant sequence/s within this fingerprint is beyond the scope of this study. Symbiont ITS2 type frequencies are shown in S7 Table.

### Analysis of the psbA^ncr^ marker

The non-coding region of the PsbA minicircle (psbA^ncr^) was amplified using custom primers and the PCR conditions shown in S2 Table. For each southern PAG site, psbA^ncr^ sequences were obtained through direct sequencing of 5 corals shown to host ITS2 type C3 in the DGGE analyses. The psbA^ncr^ sequences were aligned in MEGA6 to *S*. *thermophilum* sequences from the Gulf, in addition to other C3 types and closely related variants. A phylogenetic tree was created using the parsimony model of Mr. Bayes, with the Jukes Cantor model and gamma distribution. The MCMC was run for 2x10^6^ generations, and sampled every 1000 generations with a burn-in of 0.25. The majority 50% consensus tree imported into Dendroscope [42] for visualisation.

### Remote sensing

Monthly averaged MODIS Level 3 9km 11μm daytime SST products were downloads from the NASA Ocean Color site (http://oceancolor.gsfc.nasa.gov/). Data with flag values >0 were removed from analyses. The average maximum SST was calculated from the mean of the annual maximum of the monthly averaged SST over a 10-year period (2004–2014).

### Statistics

The composition of symbiont ITS2 types within the PAG were compared to the communities outside of the PAG (Strait of Hormuz and Gulf of Oman) using a Fisher-Freeman-Halton Exact test implemented in StatXact 11.

### Ethics statement

Coral collections were performed under permits from the Environment Agency Abu Dhabi and Fujairah municipality (United Arab Emirates), and the Ministry of Environment (Oman). No ethical approval was required for the laboratory work performed in this study.

Data available from the Dryad Digital Repository: http://dx.doi.org/10.5061/dryad.3k3m2.

## Supporting information

S1 Table Site and sampling information. Sample groups refer to classification of sites used for comparisons of symbiont communities. Mean SSTs are calculated from MODIS data as described in the Methods section.
(DOCX)

S2 Table Primers and cycling conditions for PCRs performed in this study. ^†^ Modified from a previous study [43]; ^‡^ Custom ITS region primers for Platygyra; ^*^ Custom primers to amplify psbA^ncr^ from clade C and D symbionts.
(DOCX)

S3 Table ITS2 types and associated psbA^ncr^ sequence accession numbers for sequences used from previous studies [18, 25, 44, 45] to generate the phylogenetic tree (Fig 2B). Sequences highlighted in grey belong to the *Symbiodinium thermophilum* lineage. Accession numbers for new psbA^ncr^ sequences are shown in S4 Table.
(DOCX)

S4 Table GenBank accession numbers for sequences used in this study. (DOCX)

S5 Table Haplotype frequencies for the ITS intron. (DOCX)

S6 Table Haplotype frequences for the host Pax-C region. (DOCX)

S7 Table Symbiont ITS2 types associated with individuals at each site. Each count represents the presence in an individual, including individuals that hosted mixed communities.
(DOCX)

S1 Fig Haploweb for the host ITS marker. The haplotype network (straight lines) connects haplotypes (represented as circles) based on the inferred evolutionary pathways between them. The curved lines (pink) connect haplotypes that are co-occuring in heterozygous individuals, with the thickness of the line proportional to the abundance of the heterozygous individuals. The diameter of circles representing the different haplotypes are proportional to the number of individuals that possess that haplotype and are coloured according to their relative frequency at the different sites (*Red = Delma*, *Orange = Saadiyat*, *Yellow = Ras al Khaimah*, *Green = Musandam*, *Turquoise = Fujairah*, *Blue = Muscat*).
(TIF)

S2 Fig Haploweb for the host Pax-C marker. The haplotype network (straight lines) connects haplotypes (represented as circles) based on the inferred evolutionary pathways between them. The curved lines (pink) connect haplotypes that are co-occuring in heterozygous individuals, with the thickness of the line proportional to the abundance of the heterozygous individuals. The diameter of circles representing the different haplotypes are proportional to the number of individuals that possess that haplotype and are coloured according to their relative frequency at the different sites (*Red = Delma*, *Orange = Saadiyat*, *Yellow = Ras al Khaimah*, *Green = Musandam*, *Turquoise = Fujairah*, *Blue = Muscat*).
(TIF)

S3 Fig DGGE banding profiles observed in this study. Each banding profile is labelled by the ITS2 designation above the fingerprint and shown next to its corresponding ladder (L). In cases where more than one symbiont type is present, both ITS2 designations are indicated. White arrows indicate the characteristic homoduplexes that have been extracted and sequenced previously or in this study.
(TIF)

S1 Dataset ITS and Pax-C marker phase posterior probabilities. (XLSX)

S2 Dataset Alignment of the host ITS region. (FAS)

S3 Dataset Alignment of the host Pax-C intron. (FAS)
