# Supplementary material for: Genetic structure of coral-Symbiodinium symbioses on the world’s warmest reefs
Source: PLoS One. 2017 Jun 30;12(6):e0180169. doi: 10.1371/journal.pone.0180169 (PMC5493405; doi:10.1371/journal.pone.0180169)
Supplement: S1 Table — Sample groups refer to classification of sites used for comparisons of symbiont communities. Mean SSTs are calculated from MODIS data as described in the Methods section. (DOCX) [file pone.0180169.s001.docx]

| **Site** | **Acronym** | **Location** | **Group** | **Depth** | **No. samples** | **ITS - No. haplotypes** | **PAXC – No. haplotypes** | **Mean max SST (°C)** |
| --- | --- | --- | --- | --- | --- | --- | --- | --- |
| Delma | DEL | 24.520800052.2781000 | PAG | 3m | 15 | 6 | 11 | 34.2 |
| Saadiyat | SAD | 24.599000054.4215000 | PAG | 7m | 15 | 7 | 9 | 33.8 |
| Ras al Khaimah | RAK | 25.978066656.0461167 | PAG | 2m | 14 | 8 | 8 | 33.0 |
| Musandam | MDM | 26.241153056.1972020 | Gulf of Oman | 4m | 15 | 12 | 9 | 32.3 |
| Fujairah | FUJ | 25.492946256.3635227 | Gulf of Oman | 2m | 15 | 9 | 9 | 32.3 |
| Muscat | MCT | 23.679229658.5009905 | Gulf of Oman | 3m | 14 | 11 | 7 | 31.2 |
