# Supplementary material for: Genetic structure of coral-Symbiodinium symbioses on the world’s warmest reefs
Source: PLoS One. 2017 Jun 30;12(6):e0180169. doi: 10.1371/journal.pone.0180169 (PMC5493405; doi:10.1371/journal.pone.0180169)
Supplement: S2 Table — † Modified from a previous study [43]; ‡ Custom ITS region primers for Platygyra; * Custom primers to amplify psbAncr from clade C and D symbionts. (DOCX) [file pone.0180169.s002.docx]

|  | PRIMERS | PCR CONDITIONS |  |
| --- | --- | --- | --- |
| *Host* |  | | |
| PAX-C INTRON^†^ | PAXF: 5’ACGAGGGAGGAGCTTGCKA  PAXR: 5’GGCGATTTGAGAACCAACCTGT | 94°C – 2mins  94°C – 30secs  57°C – 30secs  68°C – 60secs  68°C – 5mins | (x30) |
| ITS REGION^‡^ | YITSF: 5’AGCGTGGGATGCCGGAAAGTTGG  YITSR: 5’CCTTGCCTGATCTGAGGTCAAGAGG | 94°C – 2mins  94°C – 30secs  60°C – 30secs  68°C – 60secs  68°C – 5mins | (x30) |
|  | | | |
| *Algal Symbiont* |  | | |
| ITS REGION^[19]^ | SYM_VAR_FWD: 5’CAGCTTCTGGACGTTGYGTTGG  SYM_VAR_REV: 5’CGGGTTCWCTTGTYTGACTTCATGC | 95°C – 2mins  95°C – 30secs  56°C – 30secs  72°C – 60secs  72°C – 5mins | (x30) |
| ITS2 (DGGE)^[20]^ | SYM_VAR_5.8SII: 5’GAATTGCAGAACTCCGTGAACC  SYM_VAR_CLAMP: 5’[CGCCCGCCGCGCCCCGCGCCCGTCCCGCCGCCCCCGCCC] CGGGTTCTCTTGTTTGACTTCATGC | 95°C – 2mins  95°C – 30secs  56°C – 30secs  72°C – 30secs  72°C – 5mins | (x28) |
| psbA^ncr *^ | PSBAncr_F: 5’GGWATGGAAGTVATGCATGAAAGAAAYGC  PSBAncr_R: 5’AAGCAYCCAATRTAGAGACGATTTGYTGTGG | 98°C – 10secs  98°C – 10secs  47°C – 25secs  68°C – 135secs  68°C – 5mins | (x30) |
|  | | | |
