# Supplementary material for: Genetic structure of coral-Symbiodinium symbioses on the world’s warmest reefs
Source: PLoS One. 2017 Jun 30;12(6):e0180169. doi: 10.1371/journal.pone.0180169 (PMC5493405; doi:10.1371/journal.pone.0180169)
Supplement: S3 Table — Sequences highlighted in grey belong to the Symbiodinium thermophilum lineage. Accession numbers for new psbAncr sequences are shown in S4 Table. (DOCX) [file pone.0180169.s003.docx]

| **ITS2** | **Accession** | **ITS2** | **Accession** | **ITS2** | **Accession** | **ITS2** | **Accession** |
| --- | --- | --- | --- | --- | --- | --- | --- |
| C17a | JQ043621 | B21549 | KP280287 | BH1550 | KP280308 | BH1672 | KP280270 |
| C17a | JQ043623 | B21550 | KP280288 | BH1614 | KP280305 | BH1673 | KP280277 |
| C17 | JQ043618 | B21551 | KP280292 | BH1615 | KP280306 | BH1674 | KP280286 |
| C17 | JQ043619 | B21552 | KP280293 | BH1616 | KP280290 | BH1675 | KP280267 |
| C21a | JQ043662 | B21553 | KP280294 | BH1617 | KP280291 | BH1676 | KP280271 |
| C21a | JQ043663 | B21554 | KP280295 | BH1620 | KP280273 | BH1677 | KP280280 |
| C26a | JQ043553 | B21556 | KP280301 | BH1621 | KP280268 | BH1678 | KP280278 |
| C26a | JQ043555 | B21557 | KP280303 | BH1623 | KP280272 | BH1679 | KP280265 |
| C27 | JQ043668 | B21560 | KP280309 | BH1624 | KP280275 | BH1680 | KP280279 |
| C27 | JQ043669 | B21561 | KP280307 | BH1625 | KP280283 | BH1681 | KP280285 |
| C30 | JQ043605 | C3a | KP280231 | BH1626 | KP280259 | BH1682 | KP280284 |
| C31c | JQ043580 | C3a | KP280310 | BH1627 | KP280253 | BH1683 | KP280281 |
| C31c | JQ043601 | B335 | KP280311 | BH1628 | KP280255 | BH1684 | KP280208 |
| C31 | JQ043598 | B341 | KP280299 | BH1629 | KP280256 | BH1685 | KP280209 |
| C31 | JQ043599 | B359 | KP280223 | BH1630 | KP280257 | BH1686 | KP280207 |
| C3a | KF572399 | BH1356 | KM458293 | BH1631 | KP280248 | BH1688 | KP280266 |
| C3a | KF572400 | BH1392 | KM458294 | BH1632 | KP280244 | BH1689 | KP280210 |
| C3b | KF572386 | BH1411 | KM458273 | BH1633 | KP280249 | BH1690 | KP280212 |
| C3b | KF572387 | BH1413 | KM458274 | BH1634 | KP280245 | BH1691 | KP280216 |
| C3c | KF572299 | BH1414 | KM458275 | BH1635 | KP280246 | BH1692 | KP280211 |
| C3c | KF572300 | BH1415 | KM458276 | BH1636 | KP280242 | BH1693 | KP280218 |
| C3h | JQ043627 | BH1416 | KP280219 | BH1639 | KP280239 | BH1694 | KP280224 |
| C3h | JQ043628 | BH1417 | KM458277 | BH1640 | KP280226 | BH1695 | KP280225 |
| C3i | JQ043649 | BH1418 | KP280238 | BH1641 | KP280233 | BH1696 | KP280232 |
| C3i | JQ043651 | BH1419 | KM458278 | BH1642 | KP280235 | BH1697 | KP280241 |
| C3 | JQ043634 | BH1422 | KM458279 | BH1643 | KP280213 | BH1698 | KP280236 |
| C3 | JQ043635 | BH1423 | KM458280 | BH1647 | KP280215 | BH1699 | KP280227 |
| C3 | KF572278 | BH1424 | KM458281 | BH1649 | KP280296 | BH1700 | KP280240 |
| C3 | KF572279 | BH1425 | KM458282 | BH1652 | KP280289 | BH1701 | KP280228 |
| C3k | JQ043645 | BH1426 | KM458283 | BH1653 | KP280297 | BH1702 | KP280220 |
| C3k | JQ043646 | BH1427 | KM458284 | BH1655 | KP280298 | BH1703 | KP280234 |
| C3s | KF572380 | BH1428 | KM458285 | BH1656 | KP280304 | BH1704 | KP280221 |
| C3s | KF572381 | BH1447 | KP280217 | BH1658 | KP280302 | BH1705 | KP280229 |
| C40 | KF572357 | BH1448 | KM458286 | BH1659 | KP280300 | BH1706 | KP280230 |
| C40 | KF572358 | BH1449 | KM458287 | BH1660 | KP280262 | BH1708 | KP280222 |
| C7a | KF572222 | BH1450 | KM458288 | BH1661 | KP280263 |  | |
| C7a | KF572223 | BH1451 | KM458289 | BH1662 | KP280252 |  |  |
| C7c | KF572235 | BH1453 | KM458290 | BH1663 | KP280264 |  |  |
| C7c | KF572236 | BH1456 | KM458291 | BH1664 | KP280250 |  |  |
| C7 | KF572247 | BH1457 | KP280261 | BH1665 | KP280251 |  |  |
| C7 | KF572248 | BH1458 | KM458292 | BH1666 | KP280254 |  |  |
| C87 | KF572415 | BH1487 | KP280237 | BH1668 | KP280258 |  |  |
| C87 | KF572416 | BH1497 | KP280282 | BH1669 | KP280260 |  |  |
| Cq | KF572338 | BH1547 | KP280243 | BH1670 | KP280276 |  |  |
| Cq | KF572339 | BH1548 | KP280247 | BH1671 | KP280269 |  |  |
