# Supplementary material for: Genetic structure of coral-Symbiodinium symbioses on the world’s warmest reefs
Source: PLoS One. 2017 Jun 30;12(6):e0180169. doi: 10.1371/journal.pone.0180169 (PMC5493405; doi:10.1371/journal.pone.0180169)
Supplement: S4 Table — (DOCX) [file pone.0180169.s004.docx]

| LABEL | DESCRIPTION | ACCESSION | LABEL | DESCRIPTION | ACCESSION |
| --- | --- | --- | --- | --- | --- |
| PDITSES1 | ITS region haplotype 1 | KX026868 | PDPAXES4 | PAX-C intron haplotype 4 | KX026900 |
| PDITSES2 | ITS region haplotype 2 | KX026869 | PDPAXES5 | PAX-C intron haplotype 5 | KX026901 |
| PDITSES3 | ITS region haplotype 3 | KX026870 | PDPAXES6 | PAX-C intron haplotype 6 | KX026902 |
| PDITSES4 | ITS region haplotype 4 | KX026871 | PDPAXES7 | PAX-C intron haplotype 7 | KX026903 |
| PDITSES5 | ITS region haplotype 5 | KX026872 | PDPAXES8 | PAX-C intron haplotype 8 | KX026904 |
| PDITSES6 | ITS region haplotype 6 | KX026873 | PDPAXES9 | PAX-C intron haplotype 9 | KX026905 |
| PDITSES7 | ITS region haplotype 7 | KX026874 | PDPAXES10 | PAX-C intron haplotype 10 | KX026906 |
| PDITSES8 | ITS region haplotype 8 | KX026875 | PDPAXES11 | PAX-C intron haplotype 11 | KX026907 |
| PDITSES9 | ITS region haplotype 9 | KX026876 | PDPAXES12 | PAX-C intron haplotype 12 | KX026908 |
| PDITSES10 | ITS region haplotype 10 | KX026877 | PDPAXES13 | PAX-C intron haplotype 13 | KX026909 |
| PDITSES11 | ITS region haplotype 11 | KX026878 | PDPAXES14 | PAX-C intron haplotype 14 | KX026910 |
| PDITSES12 | ITS region haplotype 12 | KX026879 | ESPDW1 | PsbAncr - Delma sample 1 | KX026933 |
| PDITSES13 | ITS region haplotype 13 | KX026880 | ESPDW2 | PsbAncr - Delma sample 2 | KX026934 |
| PDITSES14 | ITS region haplotype 14 | KX026881 | ESPDW3 | PsbAncr - Delma sample 3 | KX026935 |
| PDITSES15 | ITS region haplotype 15 | KX026882 | ESPDW4 | PsbAncr - Delma sample 4 | KX026936 |
| PDITSES16 | ITS region haplotype 16 | KX026883 | ESPDW5 | PsbAncr - Delma sample 5 | KX026937 |
| PDITSES17 | ITS region haplotype 17 | KX026884 | ESPDS1 | PsbAncr - Saadiyat sample 1 | KX026938 |
| PDITSES18 | ITS region haplotype 18 | KX026885 | ESPDS2 | PsbAncr - Saadiyat sample 2 | KX026939 |
| PDITSES19 | ITS region haplotype 19 | KX026886 | ESPDS3 | PsbAncr - Saadiyat sample 3 | KX026940 |
| PDITSES20 | ITS region haplotype 20 | KX026887 | ESPDS4 | PsbAncr - Saadiyat sample 4 | KX026941 |
| PDITSES21 | ITS region haplotype 21 | KX026888 | ESPDS5 | PsbAncr - Saadiyat sample 5 | KX026942 |
| PDITSES22 | ITS region haplotype 22 | KX026889 | ESPDR1 | PsbAncr - RAK sample 1 | KX026943 |
| PDITSES23 | ITS region haplotype 23 | KX026890 | ESPDR2 | PsbAncr - RAK sample 2 | KX026944 |
| PDITSES24 | ITS region haplotype 24 | KX026891 | ESPDR3 | PsbAncr - RAK sample 3 | KX026945 |
| PDPAXES1 | PAX-C intron haplotype 1 | KX026897 | ESPDR4 | PsbAncr - RAK sample 4 | KX026946 |
| PDPAXES2 | PAX-C intron haplotype 2 | KX026898 | ESPDR5 | PsbAncr - RAK sample 5 | KX026947 |
| PDPAXES3 | PAX-C intron haplotype 3 | KX026899 |  | | |
