# Supplementary material for: Genetic structure of coral-Symbiodinium symbioses on the world’s warmest reefs
Source: PLoS One. 2017 Jun 30;12(6):e0180169. doi: 10.1371/journal.pone.0180169 (PMC5493405; doi:10.1371/journal.pone.0180169)
Supplement: S5 Table — (DOCX) [file pone.0180169.s005.docx]

| **HAPLOTYPE** | **DELMA** | **SAADIYAT** | **RAS AL KHAIMAH** | **MUSANDAM** | **FUJAIRAH** | **MUSCAT** |
| --- | --- | --- | --- | --- | --- | --- |
| PDITSES1 | 10 | 3 | 4 | 4 | 2 | 1 |
| PDITSES2 | 7 | 8 | 7 | 3 | 5 | 8 |
| PDITSES3 | 1 |  |  |  |  |  |
| PDITSES4 | 1 |  |  |  | 1 | 1 |
| PDITSES5 | 1 |  | 1 |  |  |  |
| PDITSES6 | 1 | 3 | 2 |  |  | 1 |
| PDITSES7 |  | 1 |  |  |  |  |
| PDITSES8 |  | 1 |  | 1 |  |  |
| PDITSES9 |  | 1 |  |  |  |  |
| PDITSES10 |  | 1 |  |  | 2 | 1 |
| PDITSES11 |  |  | 2 | 1 | 9 | 5 |
| PDITSES12 |  |  | 1 | 1 |  |  |
| PDITSES13 |  |  | 1 |  |  |  |
| PDITSES14 |  |  | 1 |  |  |  |
| PDITSES15 |  |  |  | 2 |  |  |
| PDITSES16 |  |  |  | 1 |  |  |
| PDITSES17 |  |  |  | 1 |  |  |
| PDITSES18 |  |  |  | 1 | 1 |  |
| PDITSES19 |  |  |  | 3 | 1 | 1 |
| PDITSES20 |  |  |  | 1 | 1 | 1 |
| PDITSES21 |  |  |  | 1 |  |  |
| PDITSES22 |  |  |  |  | 1 | 1 |
| PDITSES23 |  |  |  |  |  | 1 |
| PDITSES24 |  |  |  |  |  | 1 |
