# Supplementary material for: Genetic structure of coral-Symbiodinium symbioses on the world’s warmest reefs
Source: PLoS One. 2017 Jun 30;12(6):e0180169. doi: 10.1371/journal.pone.0180169 (PMC5493405; doi:10.1371/journal.pone.0180169)
Supplement: S6 Table — (DOCX) [file pone.0180169.s006.docx]

| **HAPLOTYPE** | **DELMA** | **SAADIYAT** | **RAS AL KHAIMAH** | **MUSANDAM** | **FUJAIRAH** | **MUSCAT** |
| --- | --- | --- | --- | --- | --- | --- |
| PDPAXES1 | 1 |  |  |  |  | 1 |
| PDPAXES2 | 5 | 3 |  | 2 | 2 | 2 |
| PDPAXES3 | 7 | 15 | 12 | 8 | 7 | 2 |
| PDPAXES4 | 5 | 2 | 6 | 6 | 7 | 2 |
| PDPAXES5 | 1 |  |  |  |  |  |
| PDPAXES6 | 5 | 3 | 3 | 4 |  | 11 |
| PDPAXES7 | 1 | 2 |  | 5 | 5 | 7 |
| PDPAXES8 | 1 |  |  |  | 1 |  |
| PDPAXES9 | 2 | 2 | 3 | 2 | 1 | 1 |
| PDPAXES10 | 1 |  | 1 | 1 | 1 |  |
| PDPAXES11 | 1 | 1 | 1 | 1 |  |  |
| PDPAXES12 |  | 1 | 1 | 1 | 5 |  |
| PDPAXES13 |  | 1 | 1 |  |  |  |
| PDPAXES14 |  |  |  |  | 1 |  |
