# Supplementary material for: Genetic structure of coral-Symbiodinium symbioses on the world’s warmest reefs
Source: PLoS One. 2017 Jun 30;12(6):e0180169. doi: 10.1371/journal.pone.0180169 (PMC5493405; doi:10.1371/journal.pone.0180169)
Supplement: S7 Table — Each count represents the presence in an individual, including individuals that hosted mixed communities. (DOCX) [file pone.0180169.s007.docx]

| **ITS2 TYPE** | **MUSCAT** | **FUJAIRAH** | **MUSANDAM** | **RAS AL**  **KHAIMAH** | **SAADIYAT** | **DELMA** |
| --- | --- | --- | --- | --- | --- | --- |
| A1 |  | 1 |  |  | 4 | 1 |
| C3 |  |  |  | 13 | 13 | 14 |
| C3V1 | 1 |  |  | 1 |  |  |
| C39 | 6 |  |  | 1 |  |  |
| C1V1 | 2 |  |  | 2 |  |  |
| D5 |  |  |  |  | 1 |  |
| DV1 | 7 | 14 | 15 |  |  |  |
| DV2 | 1 |  |  |  |  |  |
