# Supplementary figures and images for: Genetic structure of coral-Symbiodinium symbioses on the world’s warmest reefs
Source: PLoS One. 2017 Jun 30;12(6):e0180169. doi: 10.1371/journal.pone.0180169 (PMC5493405; doi:10.1371/journal.pone.0180169)

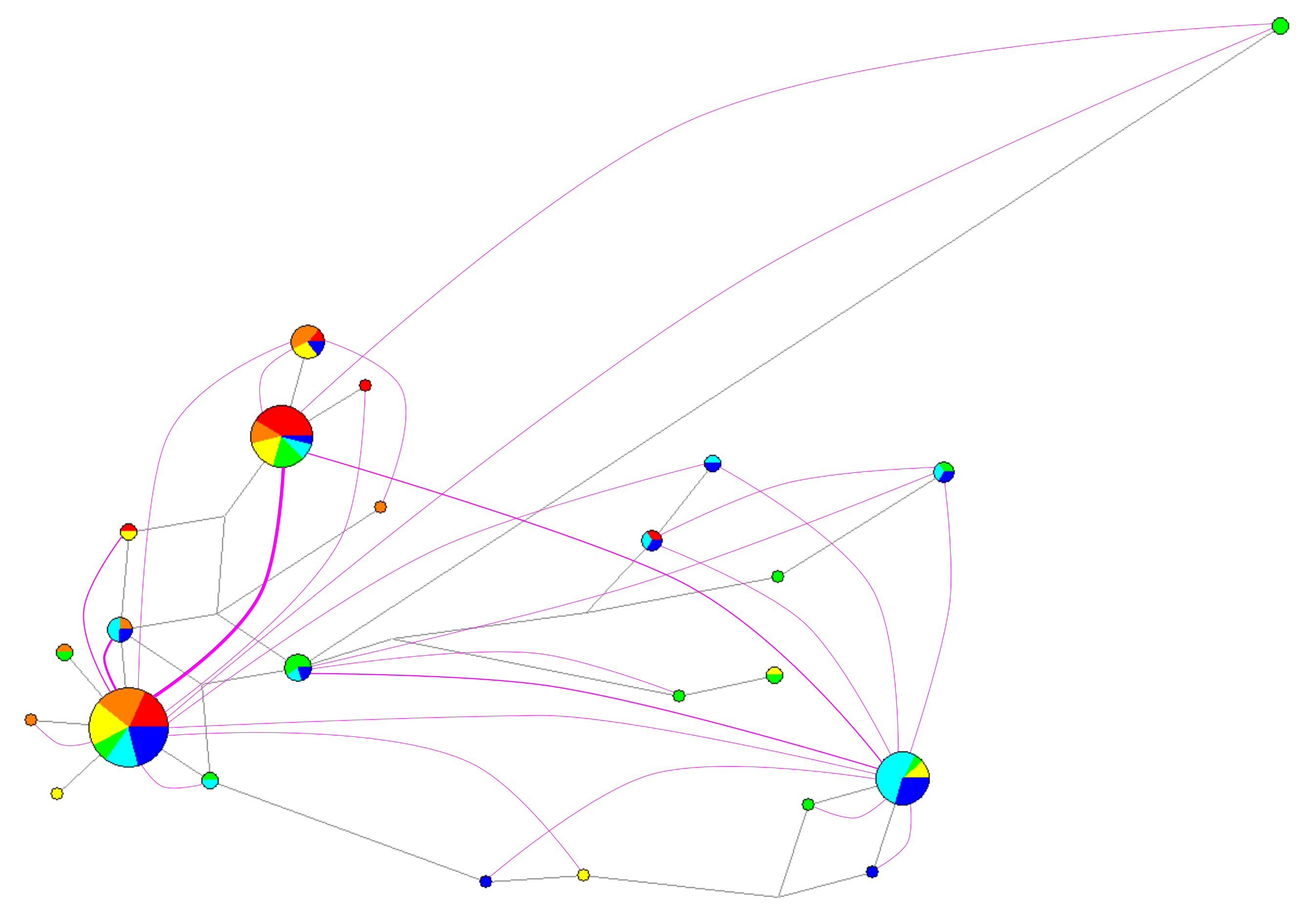

Supplement: S1 Fig — The haplotype network (straight lines) connects haplotypes (represented as circles) based on the inferred evolutionary pathways between them. The curved lines (pink) connect haplotypes that are co-occuring in heterozygous individuals, with the thickness of the line proportional to the abundance of the heterozygous individuals. The diameter of circles representing the different haplotypes are proportional to the number of individuals that possess that haplotype and are coloured according to their relative frequency at the different sites (Red = Delma, Orange = Saadiyat, Yellow = Ras al Khaimah, Green = Musandam, Turquoise = Fujairah, Blue = Muscat). (TIF) [file pone.0180169.s008.tif]

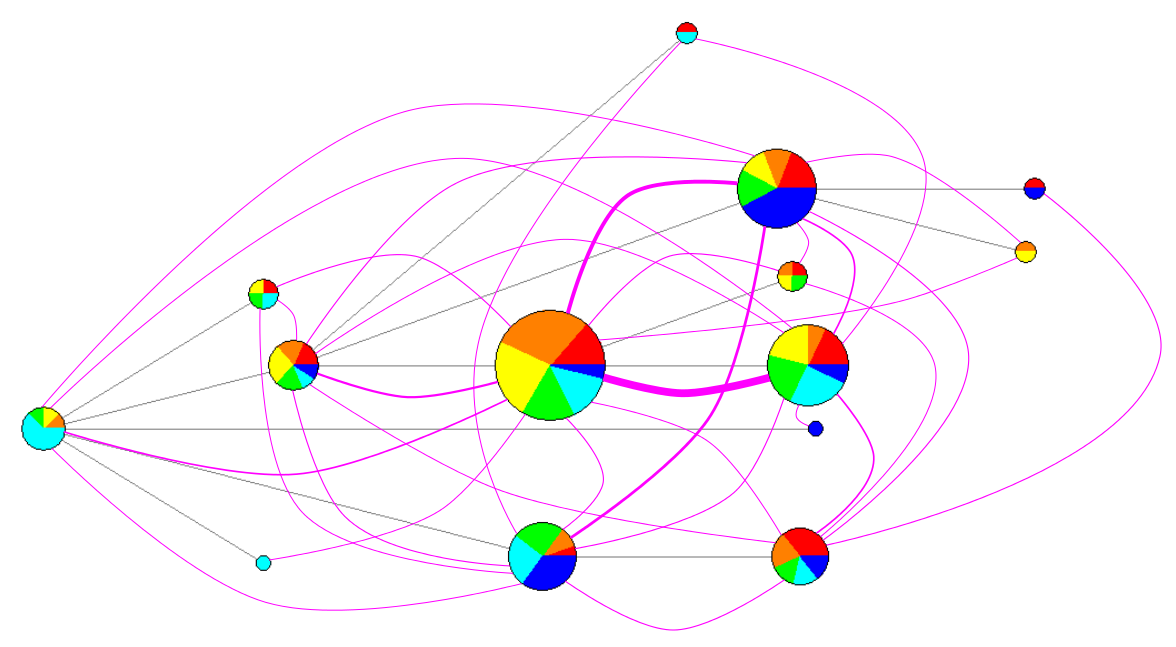

Supplement: S2 Fig — The haplotype network (straight lines) connects haplotypes (represented as circles) based on the inferred evolutionary pathways between them. The curved lines (pink) connect haplotypes that are co-occuring in heterozygous individuals, with the thickness of the line proportional to the abundance of the heterozygous individuals. The diameter of circles representing the different haplotypes are proportional to the number of individuals that possess that haplotype and are coloured according to their relative frequency at the different sites (Red = Delma, Orange = Saadiyat, Yellow = Ras al Khaimah, Green = Musandam, Turquoise = Fujairah, Blue = Muscat). (TIF) [file pone.0180169.s009.tif]

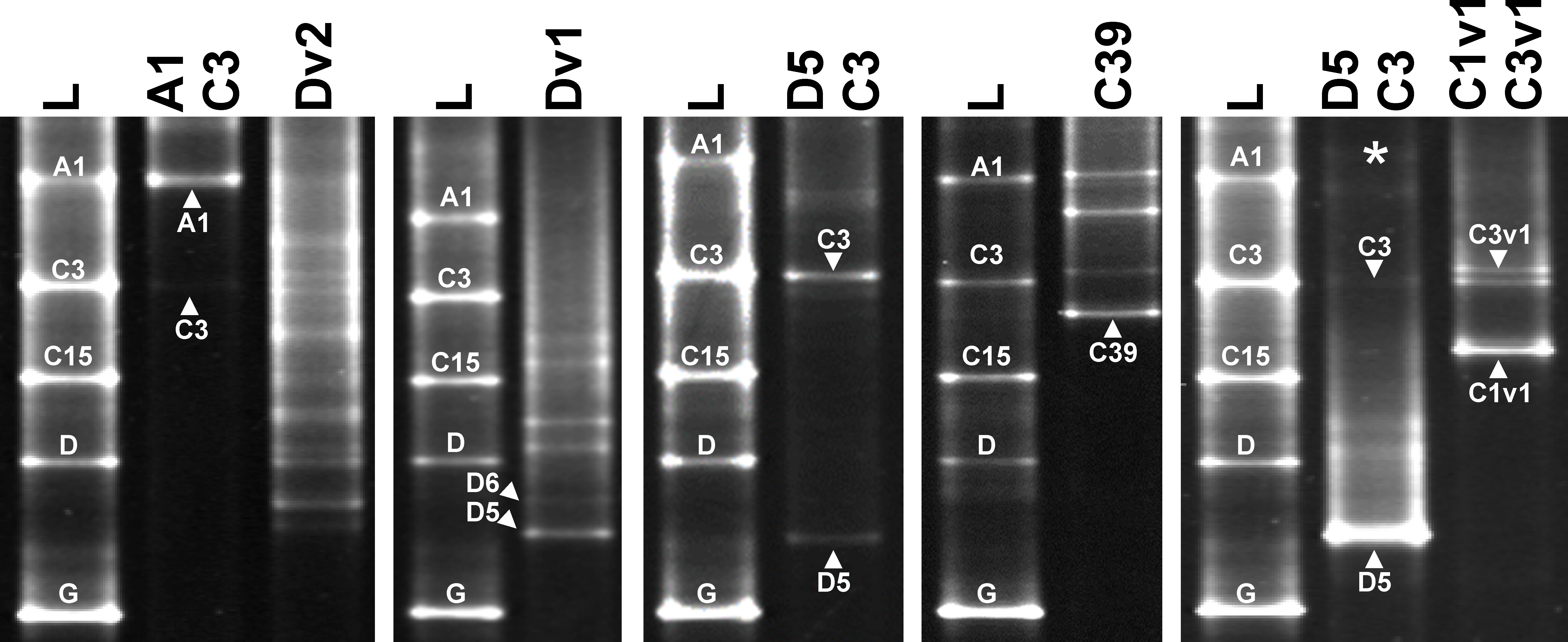

Supplement: S3 Fig — Each banding profile is labelled by the ITS2 designation above the fingerprint and shown next to its corresponding ladder (L). In cases where more than one symbiont type is present, both ITS2 designations are indicated. White arrows indicate the characteristic homoduplexes that have been extracted and sequenced previously or in this study. (TIF) [file pone.0180169.s010.tif]
